# Multiple Cerebro-Spinal Sclerosis

**Published:** 1882-07

**Authors:** 


### MULTIPLE CEREBRO-SPINAL SCLEROSIS.

On February 27th, before the Academy of Medicine, Dr. Whittaker reported the following case:

A professional gentleman, about thirty-five years of age, otherwise in robust health, was attacked with tremors about two years ago. No cause could be assigned for them; the man had been of regular habits and gave no history of syphilis. Nothing more could be learned than that he had been attacked by robbers some years ago and had received a blow on the head, but he recovered from it without any lesions. The tremors were first observed in his gait so as to excite the suspicion that he had been drinking. Gradually they invaded the upper extremities so that he was unable to write, then the head began to oscillate, and finally the tremors became general—the whole period of the symptoms covering about one year from the first onset of the disease. The tremors occurred only after muscular efforts, and then became uncontrollable. There was no defect of vision, no nystagmus.

On account of the rarity and importance in a diagnostic point of view, the speaker presented the patient to the class. When questions were put to him he answered by dividing his words into syllables—in a *scanning* measure. Some of the students recognized the case at once as one of multiple cerebro-spinal sclerosis, though one student quite naturally took it for chorea. The diagnosis is to be made between these two diseases and paralysis agitans, which is not very difficult. In sclerosis of the brain and cord the tremors are in the line of muscular action, while in chorea they are irregular and in every direction. In paralysis agitans the tremors are constant, while in sclerosis they occur only during muscular efforts. Moreover paralysis agitans generally occurs late in life, between fifty and sixty, while sclerosis belongs to adults between twenty and forty. The cause as well as pathology is obscure. All we know is that sclerotic patches are found in the brain or cord, or both, varying in size from a pin's head to a dime or even a quarter of a dollar. The disease depends on or is a chronic inflammation, but what induces it is not definitely known. An excuse for our want of more accurate knowledge of these conditions is the fact that this subject has been but recently studied, more particularly by the French and Germans. The prognosis as well as treatment is unfavorable. Nothing controls it except the constant current, chloride of barium, or nitrate of silver, and then the effect is only temporary.—*Cin. Lancet and Clinic.*

